# Elective Delivery at Term after a Previous Unexplained Intra-Uterine Fetal Death: Audit of Delivery Outcome at Tygerberg Hospital, South Africa

**DOI:** 10.1371/journal.pone.0130254

**Published:** 2015-06-15

**Authors:** Stefan Gebhardt, Leana Oberholzer

**Affiliations:** Department of Obstetrics and Gynaecology, Stellenbosch University and Tygerberg Hospital, Cape Town, South Africa; University Hospital Basel, SWITZERLAND

## Abstract

**Objectives:**

To assess the delivery outcome in a pregnancy with a previous unexplained intra-uterine death by elective induction of labour at term.

**Methods:**

An audit of the pregnancy outcome of all women within the catchment area with a current singleton pregnancy; and a previous unexplained or unexplored singleton fetal demise ≥24 weeks (or 500 grams birth weight if gestation unknown) after planned routine induction of labour at full term (39-40 weeks).

**Results:**

During the audit period, 306 patients with a previous intra-uterine fetal death were referred for further management. Of these, 161 had a clear indication for earlier intervention and were excluded from the protocol. Of the remaining 145 patients, 9 met further exclusion criteria and there were 2 patients who defaulted. Forty-two of the remaining study patients (with no known previous medical problems) developed complications during their antenatal course that necessitated a change in clinical management and earlier (<39 weeks) delivery. Of the remaining 92 patients in the audit, 47 (51%) went into spontaneous labour before their induction date; all 92 women delivered without major complications. There were no intra-uterine deaths prior to induction.

**Conclusions:**

Careful follow up at a high risk clinic identifies new or concealed maternal or fetal complications in 29% of patients with a previous intra-uterine death and no obvious maternal or fetal disease in the index pregnancy. When all risks are excluded and the pregnancy allowed to progress to full term (39-40 weeks) before an induction is offered, 50% will go into spontaneous labour.

## Introduction

The stillbirth rate for babies ≥500g is still unacceptably high in South Africa, at 42.87/1000 in the general population (for all levels of care).[1] In the relatively homogenous population served by a large referral institution (Tygerberg Hospital) in the Western Cape it was 24.5/1000 in 2011; a gradual but substantial decrease from 32.1/1000 over the last 40 years.[2] In the same year, at Tygerberg Hospital, the Perinatal Mortality (PMR) for all inborn babies ≥ 1000g was 46.6/1000; slightly higher as it is a referral hospital. In low-risk mothers with a prior stillbirth, there can be an up to 12-fold increased risk for adverse outcome in the subsequent pregnancy.[3] The biggest risk (hazard ratio 10.3, 95% confidence interval (CI) 6.1–17.2) for repeat stillbirth is before 28 weeks. In a large population based study, the adjusted odds ratio for a subsequent death was 7.1 (95% CI 3.2–15.7).[4] The risk for adverse outcome extends into the first year of life- the infant mortality rate of women with a prior stillbirth was 2.5 times higher (AHR = 2.51, 95% CI: 1.73–3.65) in a large, retrospective population-based study.[5]

Even though no studies have confirmed a fetal advantage for routine induction of labour in women with a prior stillbirth [6], in many centres, elective delivery is offered at early term (37 weeks 0 days-38 weeks 6 days) or full term (39 weeks 0 days-40 weeks 6 days). This was also the case at Tygerberg hospital, where up to 2011, women with a prior stillbirth were routinely delivered at 38 weeks 0 days.

The stillbirth rate declined slightly in the US (from 7.5 to 6.2 per 1000) from 1990 to 2003 at the same time as the rate of elective delivery before term increased; but that reduction occurred at 40 weeks or more.[7] There was no corresponding reduction in the stillbirth rate for the preterm period. As there is not currently evidence to suggest that preterm/early term labour induction will reduce the stillbirth rate, the American College of Obstetricians and Gynecologists do not currently recommend iatrogenic delivery before 39 weeks except for specific maternal or fetal conditions.[8]

Many systems used to classify stillbirths do not distinguish between cases where no cause of death was found (despite thorough investigation) and those where incomplete investigation made final diagnosis impossible. This last group is likely to represent a large proportion of unexplained stillbirths. With the use of a rigorous classification system and software (the Perinatal Problem Identification Program)[9] as well as a multi-disciplinary institutional review of every perinatal death (with post-mortem and/or placental histology where applicable), only 8.8% of stillbirths at Tygerberg Hospital are classified as of “undetermined/unknown” cause. However, in South-Africa, health care workers are often confronted with incomplete information on prior deaths and clinicians then have to accept the cause as unknown.

Tygerberg Hospital, situated in Cape Town in the Western Cape Province of South Africa, is a large regional/tertiary hospital that serves as the only specialist referral hospital (138-bed maternity unit) for the north-eastern half of greater metropolitan Cape Town. Within this catchment area (the estimated 2012 population size was 1 874 586) are eight midwife-led birthing units and three large district hospitals with emergency maternity services. The Tygerberg hospital policy on routine induction of labour for a prior stillbirth of unknown cause was changed from delivery at early term to that at full term at the end of 2011. This was necessitated in part by the large number of patients admitted for induction of labour in an already overcrowded service, with no proof of benefit. The changed hospital policy was largely based on Silver’s thorough review of the literature, which concluded that the ideal timing of delivery in most pregnancies after a prior stillbirth is at 39 weeks’ gestation. [10] A large population-based study from Scotland has shown that (at least in a developed country) elective induction of labour at term reduces perinatal mortality without an increase in the caesarean section rate. [11] The objective of this audit was to document the impact of the new protocol on pregnancy outcome in the first year after implementation.

## Materials and Methods

The study population included all patients with previous unexplained intra-uterine demise that were referred to Tygerberg hospital; in accordance with the regionalised care described in a provincial policy all women with a previous intra-uterine death identified at primary care must be referred for specialist attention. The audit period was all referrals between 1 January 2012–31 December 2012. The folders of all referred pregnant patients with a singleton pregnancy and a previous singleton fetal demise ≥24 weeks (or ≥500 gram birth weight if unknown gestation) were identified at the time of the first referral visit. Data collection was only started after clients delivered and continued until delivery of the last woman; in October 2013. All women with existing maternal or fetal disease (that may necessitate earlier delivery) at the time of the first evaluation at the high risk clinic were excluded from the hospital protocol, as they were managed clinically according to their disease profile or fetal condition; as well as women with a non-recurrent reason for the previous loss. There is a high incidence of abruptio placentae in this population, and the majority of women excluded were those with one or more previous abruptio. Patients with chronic hypertension were not excluded, as they are routinely delivered at term (40 weeks) in this institution.

The new hospital protocol aimed to reduce the burden of unnecessary intervention and did not attempt to add additional special investigations to the evaluation of a previous IUD. Following the protocol, women had a thorough history taken and attempts were made to trace all previous notes. A detail structural ultrasound was offered to all who booked before 24 weeks and a routine screening umbilical artery Doppler (reported as a Resistance Index or RI, the preferred index used in South African clinical guidelines) at 24 weeks. Women were offered a screening oral glucose tolerance test around 26–28 weeks and were followed regularly at the specialist clinic with blood pressure and urine checks and serial measurement of symphyses-fundal growth. Elective delivery was offered at term (39 weeks 0 days to 40 weeks 0 days).

As the main intention of the change in hospital protocol was to reduce the hospital stay; the primary outcome of this audit was the admission-to-discharge time. Secondary outcomes were:

Maternal:

- To assess the development of maternal complications in women with a previous unexplained/unexplored fetal demise, in particular the development of hypertension related complications and diabetes mellitus.

Fetal:

- To assess the total number of admissions of new-born neonates to the neonatal unit or intensive care unit.

Approval for the audit was obtained from the Stellenbosch University Health Research Ethics Committee (HREC S11/11/045). As it was a folder review, the HREC did not require consent to be obtained from participants. No further ethics approval was required for the analyses reported here and permission to publish was also obtained from the CEO of Tygerberg Hospital. The parent of the child depicted in the photo that accompany this manuscript has given written informed consent (as outlined in PLOS consent form) to publish the photograph.

Where appropriate, we used Chi-squared and Fisher’s exact tests for the comparison of frequencies; and the Student t test and Mann–Whitney test for comparison of means and medians, respectively. A p value of <0.05 was regarded as significant.

## Results

During the audit period, 306 patients were referred to the institution with a previous intra-uterine fetal death for further management. Of the referred patients, 161 had a definite cause for the previous IUD and they could be managed clinically according to their specific disease and were excluded from the protocol. Table 1 shows the aetiology of these previous stillbirths as identified either from a good history and/or from previous clinical notes or histology (placental or post mortem) reports.

**Table 1:** Aetiology of prior stillbirths as determined from history and/or clinical notes or available histology and post-mortem reports.

Pathology/ Disease entity	Number	Percentage
Abruptio placentae (1 or more previous abruptio)	70	43.48
Chorioamnionitis	15	9.32
Placental insufficiency (either clinical or histological)	15	9.32
Preeclampsia	13	8.07
Syphilis	6	3.73
Severe growth restriction	6	3.73
Intrapartum deaths	5	3.11
Eclampsia	4	2.48
Previous IUD related to twin pregnancy	4	2.48
Termination of pregnancy (for preeclampsia, severe IUGR, PPROM)	4	2.48
Diabetes mellitus	4	2.48
Chronic villitis	3	1.86
Trauma	3	1.86
Cord prolapse	2	1.24
Congenital abnormalities	2	1.24
Perivillous fibrin deposition with severe villitis and intervillitis	1	0.62
Chromosomal abnormalities	1	0.62
Maternal cardiac (during cardiac surgery)	1	0.62
Uterine rupture	1	0.62
Postdates	1	0.62
***Total***	***161***	***100*.*00***

Of the remaining patients, 145 were identified as having a previous unexplained or unexplored stillbirth where no definite or suspected reason could be identified. Nine patients met exclusion criteria (mostly twin pregnancies) and there were two patients that defaulted further follow up at Tygerberg Hospital.

Forty-two of the remaining 134 study patients (with no known previous medical problems) developed complications during their antenatal course that necessitated a change in clinical management and earlier (<39 weeks) delivery (depicted in Table 2). The two patients who developed abruptio placentae were respectively 28 weeks 4 days and 29 weeks and 1 day pregnant. They had uncomplicated antenatal care preceding the sudden stillbirths secondary to abruptio.

**Table 2:** Complications that necessitated earlier delivery in otherwise uncomplicated patients with previous intra-uterine fetal deaths.

Complication during antenatal course	Number	Percentage
Gestational diabetes mellitus	20	47.62
Preeclampsia	10	23.81
Placenta praevia	3	7.14
Pregnancy induced hypertension	3	7.14
Abruptio placentae	2	4.76
Severe intra-uterine growth restriction	1	2.38
Eclampsia	1	2.38
Preterm labour (PPROM)	1	2.38
Chorioangioma of the placenta	1	2.38
***Total***	***42***	***100*.*00***

**Fig 1:**
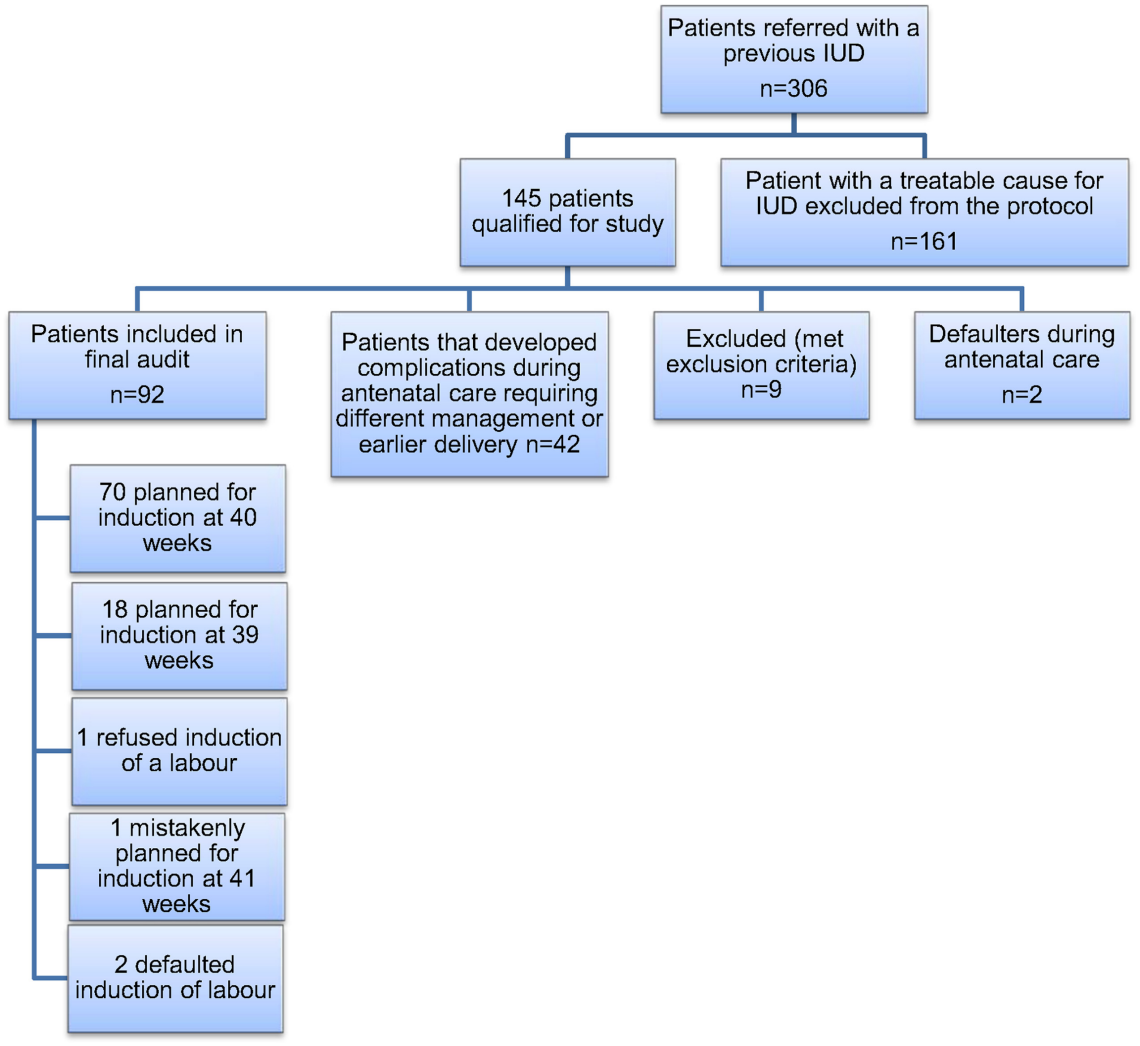
Flow diagram of participants in the study.

There remained 92 study patients with a previous unexplored/unexplained stillbirth and an uncomplicated antenatal course and they were managed according to the policy of routine induction of labour at full term. Although the protocol change was to move from routine induction at 38 weeks 0 days (early term) to full term, the decision on the exact timing of full term delivery (39 weeks/273 days or 40 weeks/280 days) was left to the attending clinician. Seventy women were scheduled for delivery at 40 weeks; and 18 for 39 weeks. Of the remainder, one patient refused induction of labour, one was planned for 41 weeks and two defaulted further management. A flow diagram of the enrolled patients is shown in Fig 1.

### Demographics

All enrolled patients come from low socio-economic circumstances; the mean age at booking was 29.4 years (range 19–41 years) and the mean gestational age at booking 17 weeks and 6 days (range: 4 weeks 2 days-37 weeks 3 days); mean gravidity 3 (range 2–7) and parity 2 (range 1–6). Most of the patients were healthy at booking. Seven patients were HIV positive and already on highly active antiretroviral treatment; five patients were known with chronic hypertension; two patients were on chronic anti-tuberculosis drugs and one was treated for known epilepsy. All women with chronic hypertension that remain otherwise uncomplicated during the pregnancy are delivered at term in this institution; therefore they could be included in the audit. A further 11 women were newly diagnosed as HIV positive at booking and started on appropriate treatment. About one-fifth of women (19.57%) indicated that they were smoking during that pregnancy. Only 13.4% of patients consumed alcohol and there were no self-acknowledged drug/substance abusers in the group.

The mean time that a client waited for referral to Tygerberg Hospital (from the date of booking until the first appointment at Tygerberg) was 31.6 days (range 1 day-255 days). The mean gestation (in days) at referral was 156 (22 weeks and 2 days) [range 6 weeks and 2 days to 39 weeks and 5 days].

Gestational age was determined according to the current provincial protocol, by correlating the dates from the last menstrual period with the early ultrasound. When certain (documented) dates correlated within one week of the sonar in the first trimester; or within 2 weeks according to the second trimester scan, the dates according to the last period was used.

When the dates were uncertain or not known, the gestational age was calculated using the earliest formal ultrasound available. All the patients underwent sonar evaluation at referral; for 73% of women (n = 66) this occurred before 24 weeks of gestation. Of the remaining 26 patients; 13 patients were referred only in the third trimester without any early ultrasound or accurate dates and the clinicians dealing with them dated them according to the late ultrasound. Ten had accurate dates that corresponded with the late ultrasound and the rest were dated according to the late ultrasound and the clinical estimate at first booking.

It is interesting to note that of the 47 patients who went into spontaneous labour, 38 booked before 24 weeks; some as early as 4 weeks; and all had detail sonographic evaluation. The other nine booked in the early third trimester and were dated according to a late ultrasound (all before 31 weeks). In fact, the gestational age as calculated by dates (and not the actual sonar EDD) were only used for 6 patients. If the gestational age is recalculated for all women using only the ultrasound expected date (and disregarding her last period), the mean gestation for spontaneous delivery in this group was still 38 weeks and 5½ days.

The hospital protocol required a screening umbilical artery Doppler test at 24 weeks; and 88 patients (95.6%) received their test. The mean gestational age at which screening Doppler was done, was 27 weeks and 3 days (range 21 weeks and 5 days to 38 weeks). Twenty-five patients (28.4%) needed a repeat Doppler for a resistance index of 75–95%. No screening Doppler was abnormal (>95^th^ centile).

The protocol further stated that a diabetic screen should be done at booking and at 28 weeks. Sixty-four patients (69.57%) had documented screening tests.

### Pregnancy outcome

Of the 92 patients included in the final audit, 47 (51.8%) went into spontaneous labour before their planned induction date. The mean gestation for onset of spontaneous labour was 272 days (38 weeks and 6 days) and the average birth weight for this group 3157.3 gram (2220g–4680g).

By sub-analysing the group, of the 70 patients who were planned for induction of labour at 40 weeks, 41 (58.5%) went into spontaneous labour and only 3 (16.6%) of the group (n = 18) planned for induction at 39 weeks. There was a difference in pregnancy outcome when induction was planned for 39 weeks versus 40 weeks- 50% of those planned for delivery at 39 weeks were eventually delivered by CS vs 22.8% of those planned at 40 weeks (p = 0.01). As can be expected, there was a significant better chance of going into spontaneous labour when the pregnancy was allowed to go to 40 weeks (Odds Ratio 6.92; 95% confidence interval 1.96–32.31; P = 0.00). This data is shown in Table 3.

**Table 3:** A breakdown of gestational age at delivery in the various groups.

	Total group (n = 92) IOL planned at term	IOL planned at 39 weeks (n = 18)	IOL planned at 40 weeks (n = 70)	p value
**Actual gestation at delivery**	39 weeks 2 days (275 days)	39 weeks 1 days (274 days)	39 weeks and 2 days (275 days)	No difference in gestation
**Birth weight at delivery: mean (and range) in grams**	3167g (2220g-4680g)	3170g (2170g-3670g)	3147 g (2220g-4680g)	No difference in birth weight
**Gestation at delivery when spontaneous onset of labour occurred**	38 weeks 6 days (272 days)	38 weeks 6 days (272 days) N = 3	38 weeks 4 days (270 days) N = 41	Odds ratio for spontaneous delivery when allowed to go to 40 weeks: 6.92 (95% confidence interval 1.96–32.31; P = 0.00)
**Gestation at delivery when elective delivery was performed**	39 weeks 6 days (279 days)	39 weeks 1 day (274 days) N = 15	40 weeks 1 day (281 days) N = 29	
**Mode of delivery**		NVD N = 9CS N = 9	NVD N = 54CS N = 16	P = 0.01

Of the 47 women planned for induction of labour but who went into spontaneous labour, only 6 were delivered with a CS; 5 for fetal distress and one for prolonged spontaneous rupture of membranes with unsuccessful attempts at induction of labour.

Induction of labour was performed in the remaining 45 patients. Twenty (44.44%) of the 45 women that were admitted for induction of labour had a caesarean delivery.

**Fig 2:**
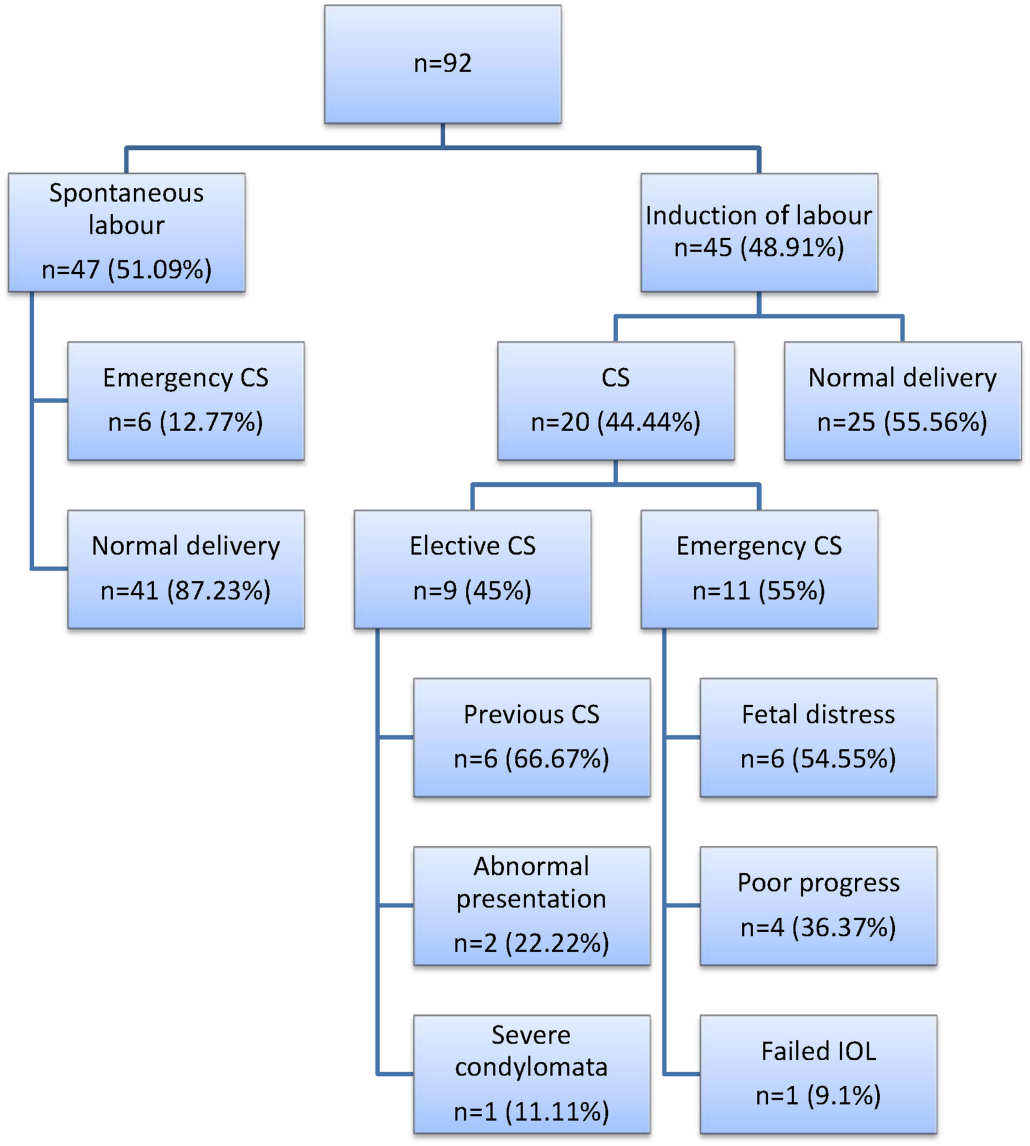
A flow diagram depicting the delivery method for the study group.

For 6 of the cases, the indication given by the attending physician was fetal compromise (fetal distress during labour). Four were due to poor progress, 1 for a failed induction and 9 were scheduled (elective) CS for obstetrical reasons. The flow diagram in Fig 2 shows the CS rate in each group as well as the reasons for CS in the induction group. There was a higher risk for any CS in the induction of labour group (RR 2.27; 95% CI 1.5–3.4; p<0.05) and also a higher risk for emergency CS in the induction of labour group (RR 1.9; 95% CI 1.16–3.1; p <0.05).

### Admission to discharge time

The median admission to discharge time (whether labour was spontaneous or not and regardless of eventual mode of delivery) for those planned for delivery at 39 weeks was 95.3 hours and for those with a planned delivery at 40 weeks was 46.7 hours (p = 0.02).

There was also a significant (p = 0.00) difference in admission-to-discharge time between the patients who went into spontaneous labour and those who underwent induction but had normal vertex deliveries.

### Neonatal outcome

All babies from the 92 cases included in the audit were born alive, with a median 5-minute Apgar score of 9 (range 8–10). Only 4 babies needed admission; the rest were discharged with their mothers. None needed ICU admission. The reasons for the admission as well as the duration of admission for these four babies are shown in Table 4. The mode of delivery is also shown.

**Table 4:** Reason for admission and duration of hospital stay for the four newborns that were admitted.

Mode of delivery	Reason for admission	Duration of admission
**Caesarean section(Elective—priorobstetrical indication)**	Transient tachypnoea of the newborn	48 hours
**Caesarean section(Breech)**	Meconium aspirationsyndrome	24 hours
**Caesarean section(Severe condylomata)**	Hydronephrosis on antenatal scan, daily monitoring of renal function until mother was discharged	72 hours
**Normal vaginal delivery**	Phototherapy	48 hours

## Discussion

This audit of pregnancy outcome after elective delivery at term showed that by postponing the delivery date with 14 days, more than half of the women would go into spontaneous labour before their elective date. There was no data available on the outcome of pregnancies before the change in induction from 38 weeks to term, so it was not possible to make a direct comparison. However, as the only change in the protocol was the planned delivery date, it can be assumed that the number of women achieving 38 weeks 0 days would have been the number receiving elective delivery before the change. If we use the outcome of the group that delivered at 39 weeks as proxy, with a CS rate of 50% and its concomitant effect on the total hospital stay (admission to discharge time), this equates to a significant burden on a busy maternity service.

This audit was not designed nor powered to measure safety in avoiding recurrence of an IUD. There was no intra-uterine death before delivery and no major neonatal complications in any of the study group. An indirect measure of safety could be the decrease in stillbirths in this population during 2011–2013 in term babies (measured as those with a birth weight of ≥2500g). The stillbirth rate in the Tygerberg catchment area for babies ≥2500g was 5/1000 during 2011; the year before the audit; and it decreased to 4.3/1000 during 2012 and further to 3.4/1000 during 2013, the last year during which patients in the audit delivered (Tygerberg Perinatal database).[9] There may be many reasons for this decrease (including a large expansion of the services with additional maternity beds during 2012), but it remain reassuring data.

The recurrence risk for IUD in this population is not known, apart from a 10% recurrence risk of stillbirth from abruptio placentae after a previous abruptio placentae. [12] For this reason, women with a history of one or more pregnancies with abruptio placentae are delivered at an earlier gestation and are specifically excluded from the hospital protocol to ensure safety.

The secondary outcome was to assess and document the development of maternal complications in women with a previous unexplained/unexplored fetal demise, in particular the development of hypertension related complications and diabetes mellitus as these are common in the hospital population and, once abruptio, congenital infection and fetal anomalies are excluded, the most important associated factors in stillbirths. It was interesting to note that, even with the protocol violation (not all women were screened for diabetes as instructed) almost one third of women selected to be otherwise well (apart form a history of one previous unexplored/unexplained stillbirth) developed signs of hypertensive disease or diabetes during antenatal care. For this reason, women with a previous IUD remain at risk and should preferably be managed at a specialist referral hospital.

The other potential cause of recurrent late stillbirth is idiopathic late-onset intra-uterine growth restriction (IUGR). Allowing women with a previous unexplained IUD (that may have been due to undetected IUGR) to proceed to term before offering elective delivery may theoretically exposed them to a risk of recurrent IUGR and fetal death before term. At Tygerberg, stillbirth from idiopathic IUGR is relatively rare- 0.17% of all deliveries ≥500g; and specifically 6.4% of all the stillbirths in the weight category ≥2000g. This weight group can reasonable be taken as IUGR developing in, or persisting to, later gestation. The majority of babies dying from idiopathic IUGR in the Tygerberg database were from primigravid mothers, and 38% of mothers had patient-related preventable factors (never booked or did not respond to decreased fetal movements). They will be managed accordingly in the subsequent pregnancy and not be included in the protocol for delivery at term; and we can be reasonably sure that the potential number of women with a previous ‘unexplained’ stillbirth due to a missed diagnosis of late onset IUGR will be small. The only screening for IUGR in the current setting is a Doppler RI of the umbilical artery at 24 weeks and serial symphysis-fundus growth measurements, with repeat Dopplers and growth scans if there is measured growth below the 10^th^ centile for this population. Future studies can focus on the actual number of women with late onset IUGR in the ‘unexplained’ group, but large numbers of study women will be needed.

The findings of this audit compares to a retrospective cohort study of Black et al [13], who documented the obstetric outcomes in second pregnancies in a large group of women, using those with an IUD in their first pregnancy as the study cohort. Women with a prior IUD (all causes) had an increased risk of pre-eclampsia and placental abruption, but the adjusted odds ratio for recurrent stillbirth was not significant (1.2 and 95% CI 0.4–3.4). When a subset of women; those with a previous unexplained intrauterine death (44% of all stillbirths) were adjusted for pre-eclampsia, abruption, preterm delivery and low birth weight, there was not a significant higher incidence of recurrent loss. Bhattacharya et al [14] extended the sample size of this same population nine-fold in an attempt to improve the power, but could not repeat the analysis for unexplained stillbirths, as information on the cause of stillbirth was not available.

## Conclusions

A pregnant woman who had an unexplained stillbirth previously is at risk of developing medical or fetal complications during pregnancy and should be managed at a specialist referral unit. If the pregnancy is otherwise uncomplicated, an elective delivery planned at 40 weeks gestation reduces the number of admissions for induction of labour as well as the overall hospital stay.
